# Evaluation of a Computer-Based Morphological Analysis Method for Free-Text Responses in the General Medicine In-Training Examination: Algorithm Validation Study

**DOI:** 10.2196/52068

**Published:** 2024-12-05

**Authors:** Daiki Yokokawa, Kiyoshi Shikino, Yuji Nishizaki, Sho Fukui, Yasuharu Tokuda

**Affiliations:** 1 Department of General Medicine, Chiba University Hospital, Chiba, Japan; 2 Department of Community-Oriented Medical Education, Chiba University School of Medicine, Chiba, Japan; 3 Division of Medical Education, Juntendo University School of Medicine, 2-1-1 Hongo, Bunkyo-ku, Tokyo, 113-8421, Japan, 81 3-3813-3111, 81 3-5689-0627; 4 Department of Emergency and General Medicine, Kyorin University, Tokyo, Japan; 5 Immuno-Rheumatology Center, St. Luke’s International Hospital, Tokyo, Japan; 6 Division of Rheumatology, Brigham and Women’s Hospital, Boston, MA, United States; 7 Muribushi Okinawa Center for Teaching Hospitals, Okinawa, Japan; 8 Tokyo Foundation for Policy Research, Tokyo, Japan

**Keywords:** General Medicine In-Training Examination, free-text response, morphological analysis, Situation, Background, Assessment, and Recommendation, video-based question

## Abstract

**Background:**

The General Medicine In-Training Examination (GM-ITE) tests clinical knowledge in a 2-year postgraduate residency program in Japan. In the academic year 2021, as a domain of medical safety, the GM-ITE included questions regarding the diagnosis from medical history and physical findings through video viewing and the skills in presenting a case. Examinees watched a video or audio recording of a patient examination and provided free-text responses. However, the human cost of scoring free-text answers may limit the implementation of GM-ITE. A simple morphological analysis and word-matching model, thus, can be used to score free-text responses.

**Objective:**

This study aimed to compare human versus computer scoring of free-text responses and qualitatively evaluate the discrepancies between human- and machine-generated scores to assess the efficacy of machine scoring.

**Methods:**

After obtaining consent for participation in the study, the authors used text data from residents who voluntarily answered the GM-ITE patient reproduction video-based questions involving simulated patients. The GM-ITE used video-based questions to simulate a patient’s consultation in the emergency room with a diagnosis of pulmonary embolism following a fracture. Residents provided statements for the case presentation. We obtained human-generated scores by collating the results of 2 independent scorers and machine-generated scores by converting the free-text responses into a word sequence through segmentation and morphological analysis and matching them with a prepared list of correct answers in 2022.

**Results:**

Of the 104 responses collected—63 for postgraduate year 1 and 41 for postgraduate year 2—39 cases remained for final analysis after excluding invalid responses. The authors found discrepancies between human and machine scoring in 14 questions (7.2%); some were due to shortcomings in machine scoring that could be resolved by maintaining a list of correct words and dictionaries, whereas others were due to human error.

**Conclusions:**

Machine scoring is comparable to human scoring. It requires a simple program and calibration but can potentially reduce the cost of scoring free-text responses.

## Introduction

The General Medicine In-Training Examination (GM-ITE) tests clinical knowledge in a 2-year postgraduate residency program in Japan. It uses a method similar to that of the Internal Medicine Residency Examination in the United States [1]. Its purpose is to improve training programs by providing residents and program directors with an objective and reliable assessment of clinical knowledge [2]. The validity of the GM-ITE has been confirmed in previous studies [3]. Currently, the GM-ITE is taken optionally by each training hospital; approximately one-third of residents take the exam each year [4].

The GM-ITE also includes video and audio questions with the potential to capture nonverbal expressions and communication. In the academic year 2021, the Japan Institute for Advancement of Medical Education Program (JAMEP) introduced questions using a patient simulation video. As a domain of medical safety, the video-based questions were designed to evaluate the diagnosis based on a patient’s medical history and physical findings and the competency in presenting the case appropriately. All previous 80 GM-ITE questions for assessing clinical knowledge were multiple-choice questions because of the large number of residents who took the exam. In handover assessments, problem representation and diagnostic justification are essential skills in clinical reasoning [5]. However, as multiple-choice questions do not measure these skills, essay-style questions are appropriate [5]. To have participants provide the handover text, the video-based question asked for free-text answers by digital text input. With the introduction of digital tests, computer-based tests may require free-text answers [6]. Assessments using free-text responses in medical education can facilitate qualitative evaluations and be used to evaluate clinical reasoning in Objective Structured Clinical Examinations [7,8]. However, in Japan, the GM-ITE is the only test that requires free-text responses for medical professionals.

In several studies, scoring free-text responses required 1‐3 minutes per question [8,9]. As the GM-ITE becomes widespread, more time is needed for scoring, which is a major barrier to implementing questions with a free-text response format. Given the future considerable increase in the number of test takers, automatic scoring is necessary to ensure fairness in the scoring process, speed up the checking of answers, and reduce the time, cost, and human effort.

Therefore, this study aimed to create a program for scoring free-text responses and verify its validity by comparing it with human scoring. There are no reports of the automatic scoring of free-text responses in examinations administered to physicians or residents. Some studies have reported automatic scoring systems for free-text responses for several languages and models, including complex models such as Long Short Term Memory and Bidirectional Encoder Representations from Transformers with a large amount of training data [10]. As the free answers used in this study can be broken down into words, a simple model is sufficient, as scoring is possible by matching words from the answers to a prepared list of correct answers.

## Methods

### Participants

Physicians who took the GM-ITE had the option to answer patient reproduction video-based questions with simulated patients as a trial exam outside of the scoring of the original exam. We used free-text response data for the video-based questions that the first- and second-year postgraduates responded to. The questions covered contained patient-replicated video-based Parts 3‐6 (Table 1). The questions were based on a 5-minute video of a 45-year-old male patient who had deep vein thrombosis and subsequent pulmonary thromboembolism following a lower extremity fracture. Participants responded with short digital sentences containing the correct concept in 100 words or less. The patients were briefed as follows: After completing the medical interview and physical examination, you decided to hand this patient over to the internal medicine physician, who would oversee admission from the planning stage of the examination. Create the transfer for this patient according to the Situation, Background, Assessment, and Recommendation (SBAR) [11,12]. We excluded all the nonresponsive cases from the data.

**Table 1.** Questions, concepts, and sample answers.

Parts	Questions	Question number	Concepts in the question	Sample answers
3	Describe the situation in 100 words.	3‐1 3‐2 3‐3 3‐4 3‐5	Name, age, sex, provisional diagnosis, and chief complaint	The patient is Seiichi Yoshinaga, a 45-year-old male visiting the emergency room with his wife because he had a syncope at home last night. It was his first syncope and he was admitted to the hospital to investigate the cause. I consider acute pulmonary thromboembolism as a tentative diagnosis.
4	Describe the background in 100 words.	4‐1 4‐2 4‐3 4‐4	History, medications, the state when he lost consciousness, and duration	His medical history includes a fracture in the right lower leg. He is currently taking no medications. According to his wife, an eyewitness, he blacked out at his home and recovered within a few minutes. There were no convulsions.
5	Provide an assessment in 100 words.	5‐1 5‐2 5‐3 5‐4 5‐5	Tachypnea (external), jugular vein distention, diastolic murmur, increased pulmonary artery component of S2, and bone fracture	Physical examination reveals tachypnea, jugular vein distension, systolic murmur, and increased IIp^a^. Given the episode of syncope from a fracture in the right lower leg, I consider that he has acute pulmonary thrombosis.
6	Provide a recommendation in 100 words.	6‐1 6‐2 6‐3 6‐4 6‐5	Electrocardiogram, cardiac ultrasound, computed tomography (CT) with contrast, anticoagulant drugs, and thrombolytic drugs	The plan includes imaging studies with electrocardiogram, echocardiography, and contrast-enhanced CT. In addition, I would start anticoagulants and thrombolytic therapy based on the results of these tests.

a IIp: The pulmonic closure sound component of the second heart sound.

### Human Scoring

The scorer judged whether the free-text responses obtained for each part contained the concepts listed in Table 1. They added 1 point for each of the concepts included in the response, for all parts. The final score (human) was obtained by matching the scores obtained by 2 independent scorers. The scorers have experience in creating and evaluating GM-ITE exam questions, and their scoring skills are standardized. The scorers received thorough training beforehand and evaluated the responses based on consistent criteria. If 2 scorers gave different scores, 4 people (including these 2) met to reconcile the differences.

### Machine Scoring

Machine scoring is the method proposed for this project. We used a simple model and created a list of words indicating the concept of the correct answer. The machine judged whether the free-text responses included the words in the list. For question 4‐3, “the state when he lost consciousness” indicates the correct concept; these words are needed to form a sentence of a certain length. Therefore, we were unable to generate a list of words for 4‐3 and excluded it from the study.

### Preparing a Correct Word List

Multimedia Appendix 1 lists the correct words for each question. We added several words to the list to accommodate synonyms and notation fluctuations (eg, Arabic, Roman, and Chinese numerals). For all questions, including questions 3‐1 “name,” 3‐4 “provisional diagnosis,” and 4‐2 “medications,” which are proper nouns, misspelled words were marked as wrong answers, given that the questions were intended to convey information reliably.

### Response Preprocessing

We converted the free-text responses into word sequences to match the words in the same sequence as the prepared list. Because there is no space between words in Japanese, it was necessary to split sentences based on a prepared dictionary or specific algorithm. We performed segmentation and morphological analysis of the response texts to produce word sequences converted into the standard form. We used MeCab [13] for the segmentation and morphological analyses. We used mecab-ipadic-NEologd [14], ComeJisyo [15], and a user dictionary (described in the next section). ComeJisyo is a dictionary for MeCab that collects terms used in medical facilities.

### Dictionary Registration of Overdivided Words

MeCab cannot handle words that are not in the dictionary (ie, unknown words), which are sometimes split into smaller (than expected) parts in the dictionary, by word. Consequently, the words in the free-text responses may not match the prepared word list exactly, and the machine may judge them as incorrect answers. For example, the noun “Yoshinaga Seiichi” is divided into “Yoshinaga” and “Seiichi,” and “ultrasound” is divided into “ultra” and “sound.” Words are also divided into parts when they include a particle. For example, “*ishiki* (consciousness) *wo* (particle) *ushinau* (lose)” is split into “consciousness,” “particle,” and “lose.” Therefore, we prepared a user dictionary (Multimedia Appendix 2) to avoid the overdivision of nouns. We also considered a verb to be in the form containing a particle and registered it in the user dictionary, including its conjugated form. We set 1 as the “cost,” parameter of MeCab, of all words added to the dictionary.

### Calculating Score (Machine)

In this study, we defined “score (machine)” as the score computed by the machine-scoring model. The machine scoring method is described as follows: (1) The machine extracts the free-text response of Part 3 from the subject’s answers; (2) It preprocesses the extracted text into a sequence of words separated by spaces; (3) For question 3‐1 in Part 3, it extracts a word from the correct word list and checks whether the sequence includes the word; (4) If there is more than 1 word in the word list for the same question, it checks all other words individually; (5) It repeats this check for the question, and if there is at least 1 word in the word list in the answer, it awards 1 point for that question; (6) It repeats the steps above for all parts and all questions; (7) After scoring one subject, it repeats the steps above for all subjects.

### Analytics

We calculated the human and machine scores for Parts 3‐6 for all subjects. We set the null hypothesis that the population of these scores be equal and performed a Mann-Whitney *U* test. Note that Q3, Q5, and Q6 took values from 0 to 5; Q4, from 0 to 3; and the total score, from 0 to 18. We performed the analysis in the terminal whose operating system was Ubuntu 20.04.2 LTS, the CPU was an AMD Epyc 7402 p 24-core processor, and the primary memory was 256 GB. We used Python (3.6.9). Data were analyzed using IBM SPSS Statistics (version 29.0.2.0; IBM Corp).

### Ethical Considerations

This study was approved by the Ethics Review Board of the Japan Institute for Advancement of Medical Education Program (approval number 21‐10). Informed consent was obtained from all the participants on the computer, and only those who provided consent were included in the study.

## Results

We collected 104 responses—63 for postgraduate year 1 and 41 for postgraduate year 2. We excluded 27 ongoing response data and 38 nonresponse data, with all null values for Q3-Q6, leaving 39 cases for analysis. The median response time was 20 minutes 24 seconds (Q1-Q3: 16 min, 12 s to 31 min, 16 s). The average execution time of 10 runs of the program (main.py) for the 39 cases was 0.638 seconds (SD 0.007 s).

**Figure 1.**
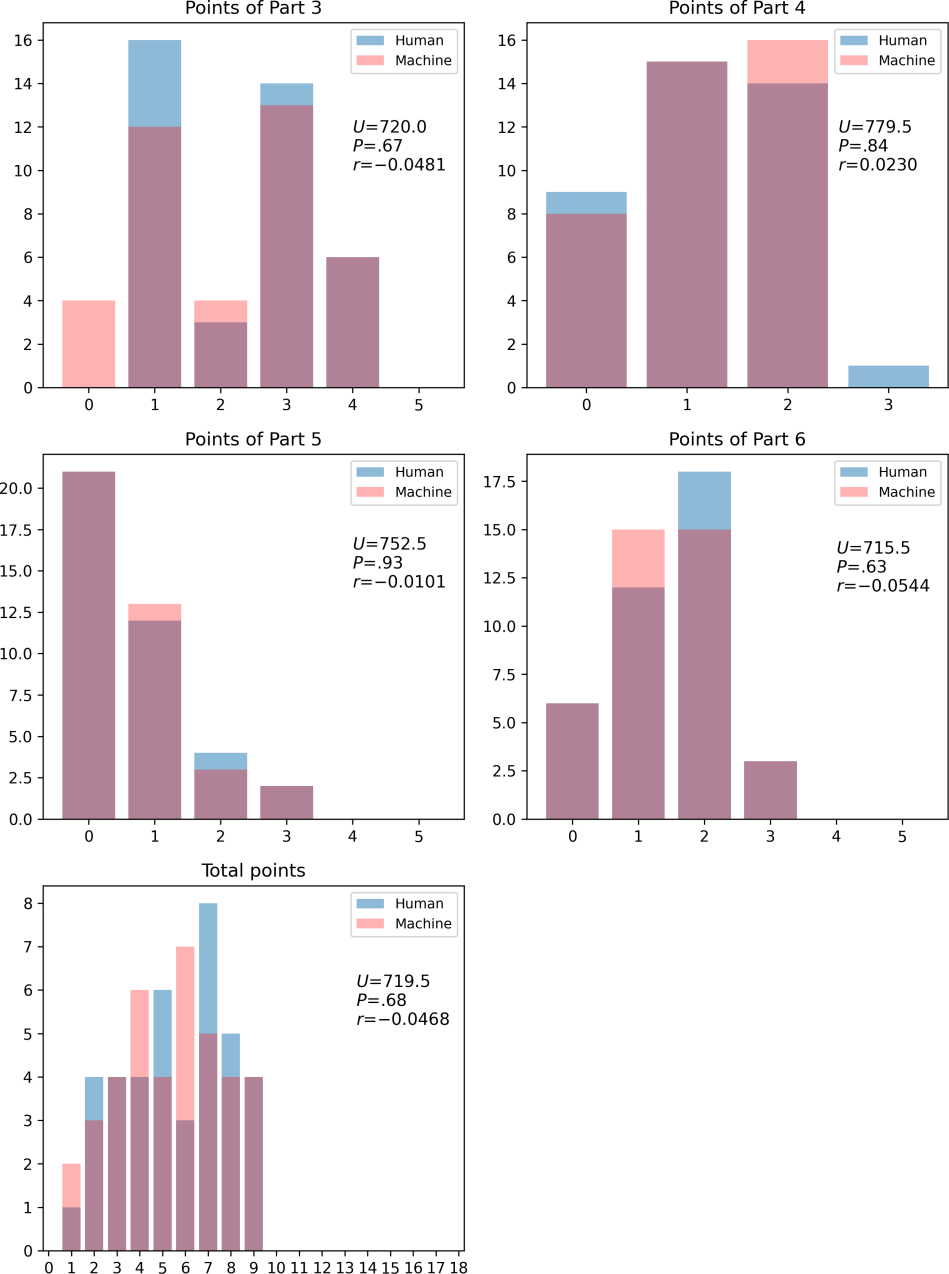
Histogram of human and machine scores. *U*, *P*, and *r* indicate the *U* statistic, *P* value of the Mann-Whitney *U* test, and effect size, respectively. The effect size *r* is calculated as *r*=Z/sqrt(n), where Z is the standardized test statistic.

Figure 1 shows the number of points obtained for each question separately for human and machine scores. We did not reject the null hypothesis in the Mann-Whitney *U* test for the total score and Parts 3‐6.

There were 265 answers (number of questions × number of subjects = 5 questions × 39 cases). Fourteen questions (7.2%) had discrepancies between the human and machine scores (Table 2). Of the questions judged correctly by humans, 11 were judged incorrectly by machines. Multimedia Appendix 3 shows the answers to Table 2 in Japanese and English.

We created a cross table (Table 3) to compare the accuracy of human and machine scoring based on the total scores. Each question had a different maximum score: Q3 had a maximum of 5 points, Q4 had 3 points, Q5 had 5 points, and Q6 had 5 points, for a total possible score of 702 points (18 points × 39 participants = 702 points). Of the 702 total points, 206 were judged correct by both the machine and human, and 482 were judged incorrect by both. Of the 14 discrepancies shown in Table 2, there were 3 false positives (cases where the machine judged correctly, whereas the human judged incorrectly) and 11 false negatives (cases where the human judged correctly, whereas the machine judged incorrectly). The accuracy was 0.980, the recall was 0.949, the precision was 0.986, and the *F*_1_ score was 0.967.

The table summarizes the comparison between human and machine scoring. The 3 false negatives correspond to Table 2 entries #6, #8, and #9, while the 11 false positives correspond to entries #1‐5, #7, and #10‐14.

**Table 2.** Analysis of discrepancies between human and machine scoring.

ID	Question number	Answers in English (complete sentence)	Human	Machine	Reason for the discrepancy
#1	3‐5	He is a 45-year-old male who collapsed with a disturbance of consciousness^a^ without signs.	Correct	Mistake^b^	“Disturbance of consciousness” is an incorrect answer.
#2	3‐5	He was plunged into darkness and fell.	Correct^b^	Mistake	This is a sentence.^c^
#3	3‐5	This patient fell after feeling^d^ dimmed vision in the restroom.	Correct	Mistake^b^	The correct word list does not include “dimmed vision”; it is also misspelled^d^.
#4	3‐5	He stood up to go to the restroom and fell. He had a dimmed vision.	Correct^b^	Mistake	The correct word list does not include “dimmed vision,” and this is a sentence^c^.
#5	3‐5	When he stood up, he was plunged into darkness and fell and	Correct^b^	Mistake	This is a sentence^c^.
#6	4‐1	He fell and was noted to have a fracture near his right ankle joint.	Mistake	Correct^b^	Human error
#7	4‐1	He has an injury to his right leg, which is in a cast.	Correct	Mistake^b^	“Injury”^a^ is an incorrect answer.
#8	4‐2	Medications: Amlodipine	Mistake	Correct^b^	Human error
#9	4‐2	He has hypertension and takes Amlodipine.	Mistake	Correct^b^	Human error
#10	4‐3	He had a blackout, which improved quickly.	Correct^b^	Mistake	“Improved quickly” is not in the dictionary.
#11	5‐2	Valvular disease is also a differential based on jugular^d^ vein distension and systolic murmur.	Correct	Mistake^b^	Misspelled^d^
#12	6‐2	Echography is first used to deny pulmonary embolism and transient cerebral ischemia.	Correct	Mistake^b^	“Echography”^a^ is an incorrect answer.
#13	6‐2	I should perform an echo to evaluate valve motion.	Correct	Mistake^b^	“Echo”^a^ is an incorrect answer.
#14	6‐2	He needs a close examination, including an echo, to see if the EF^e^ is preserved.	Correct	Mistake^b^	“Echo”^a^ is an incorrect answer.

a The answer is incorrect, not on the list of correct answers, or not included in the dictionary.

b It is a correct decision.

c Original Japanese sentence cannot be broken down into words and is expressed as a sentence.

d Indicates a misspelling.

e EF: ejection fraction.

**Table 3.** Confusion matrix comparing human and machine scoring.

	Machine scoring		
	Correct	Mistake	Total
**Human scoring**			
Correct	206	11	217
Mistake	3	482	485
Total	209	493	702

## Discussion

### Principal Findings

This pilot study showed no statistically significant difference between human and machine scoring using a simplified model of free-text responses on the GM-ITE’s patient reproduction video-based questions with simulated patients. As numerous physicians take the GM-ITE, the time cost of scoring free-text responses is high. The time required to analyze 39 cases was less than 1 second, which significantly reduced the time and human costs. Furthermore, there were no statistical differences in the scores, and the reasons for these discrepancies were clear. Therefore, this model can expand the number of participants and address other problems while maintaining a satisfactory level of accuracy.

In the 14 cases where the human and machine scores differed, 11 questions that humans scored as correct were judged incorrectly by the machine. Therefore, in general, the human scores were higher. It is necessary to examine whether this result overestimates human scoring or underestimates machine scoring. There are at least 6 reasons for these differences, discussed below.

### Instances Where the Correct Word List Did Not Include Human-Deemed Correct Words

In Table 2, #4 and #10 fit this category. Although the word “dimmed vision” was correctly separated by spaces, it was not in the correct word list (Multimedia Appendix 1). The machine failed to match it and judged it as incorrect. In such cases, we can improve the machine’s scoring accuracy if the exam preparer considers the correct word when preparing the answer.

### Instances Where Humans Read the Sentence and Judged It as Correct

In Table 2, #2, #4, #5, and #10 fit this category. For example, in #2, “He was plunged into darkness and fell,” the scorer understood that the respondent described a dimmed vision or loss of consciousness. However, it is challenging to prepare sentences in dictionaries. Meanwhile, #4 “He stood up and fell” suggests an episode of fainting due to orthostatic hypotension, but the machine cannot recognize this answer as correct.

**Figure 2.**
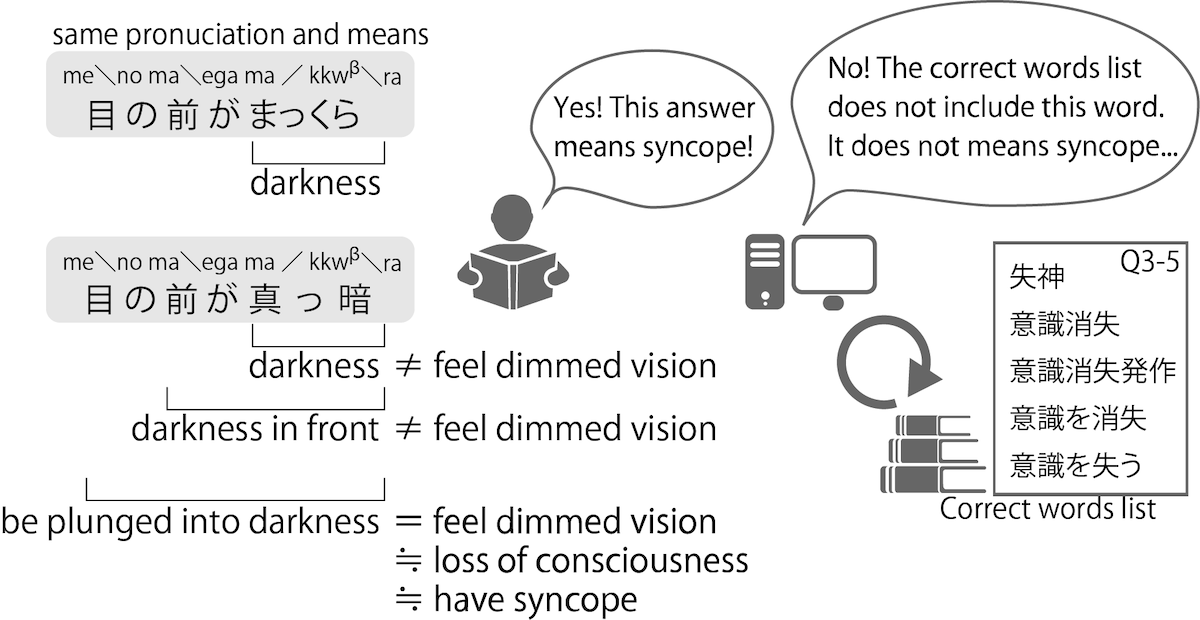
Japanese sentences similar to “syncope.” These 2 Japanese sentences with different notations read the same and indicate the same meaning. The meaning changes depending on the position at which the sentence is separated.

In Japanese, sentences can be expressed using a mixture of hiragana, katakana, Chinese characters, and the alphabet. Furthermore, there is no strict word order. It is possible to interchange hiragana and Chinese characters to create sentences that make sense. For example, in Figure 2, these 2 Japanese sentences have the same pronunciation and meaning of syncope. Humans read the sentence, understand it, and judge it as the same. However, machines understand that these sentences are different.

The algorithm used in this study is simple and does not assume the parsing of sentences. We require another system that uses deep learning to understand the meaning of sentences. In general, systems that use deep learning incur high modeling and training costs. Rather than building such a system, it would be less expensive to continue using a simple algorithm by restricting the response method. It is possible to guide the respondents to words by including a sentence “Answer in words, not a sentence” or “Answer using medical terms” in the question beforehand or by imposing a character limit on the number of words in the answer box. By combining words to give the correct answer, the number of question variations can be increased.

### Instances Where a Word Humans Judged as Correct Was Incorrect

In Table 2, #1, #7, #12, #13, and #14 fit this category. In #1, humans ruled “disturbance of consciousness” as valid; however, the machine did not because the correct word list (Multimedia Appendix 1) included “loss of consciousness” and “syncope” but not “disturbance of consciousness.” Similarly, the list does not contain the word “injury,” but “fracture.” Simply including “disturbance of consciousness” or “injury” in the correct word list does not solve this problem. In this context, the word “disturbance of consciousness” is not medically correct. “Injury” is also less informative and may not be appropriate in the context of a transfer of information. Therefore, in these cases, the human judged the incorrect answer to be correct.

Regarding the word “echo,” it is unclear where and what type of echo examination is required. Even if the context refers to “echocardiography,” the respondent did not clearly state the echo type. This is inappropriate for use as an informative sentence. If the sentence was “to check valve motion by echo” or “to check for preserved EF by echo,” it could be addressed by including “valve motion” or “EF” as the correct word.

### Instance of Oversegmentation of a Correct Word Due to Dictionary Mismanagement

In Table 2, #10 fits this category. “Improved quickly,” which is “*sugu* (quickly) *ni* (particle) *kaizenshita* (improved),” is a sentence, not a word. Our algorithm splits it into “quickly,” “particle,” and “improved.” We can include the words in both the dictionary and the correct word list by improving the dictionary to avoid splitting.

### Cases of Misspelling

In Table 2, #3 and #11 fit this category. In a previous meeting, the exam preparer decided that the spelling was incorrect. Nevertheless, human scorers were unable to recognize the misspelling. In other words, these correct decisions are the result of rigorous machine scoring. As the participants answered with digital data, it is common to mistakenly input homonymic Chinese characters. This problem is a combination of 2 factors—the respondent’s error and the error of the human who scored the answer. In these cases, machines can score rigorously.

### Cases of Human Errors

In Table 2, #6, #8, and #9 fit this category. Although the words “fracture” and “amlodipine” were available as correct answers, human scorers could not judge them as correct. These correct decisions were the result of machine scoring.

We found that machine scoring was underestimated in 4 out of 14 cases, whereas human scoring was overestimated in 10 out of 14 cases. Machine scoring is not necessarily inferior to human scoring. Efforts to reduce variances would be required. Discrepancies that depend on questions should be eliminated by calibration against human scoring before machine scoring implementation. Calibration is essential for the current system, and the cost is lower than that of the human scoring of thousands of responses. This study can be interpreted as 39 calibrations for several thousand machine scores.

This algorithm can cope with the increase in the number of examinees using machine scoring. Annually, approximately 9000 individuals take the GM-ITE, and the algorithm employed in this study demonstrates its generalizability. Such a format allows for the presentation of a wide range of problem variations and enables the evaluation of diverse domains. This method can also be adapted for examinations, not only for physicians and residents but also for other health care professionals. In Japan, the common achievement tests are publicly administered, including computer-based tests (CBTs) that fourth-year medical students take. Moreover, the Japan Association of Nursing Programs in Universities is implementing a project to introduce a common achievement test of nursing and demonstrate a prepractice CBT system [16]. In Japan, around 50,000 people pass a new national nursing exam annually [17]. In addition, around 2000 dentists and 10,000 physical therapists pass the exam annually. In the future, examinations to qualify and assess the competence of health care professionals should be conducted using CBT.

### Future Directions

This pilot study focused on a Japanese examination. Japanese includes multiple character types such as hiragana, katakana, and kanji, and lacks spaces between words, making it more complex for natural language processing compared with other languages. However, the success of this scoring program for Japanese indicates its potential applicability to other languages for the following three reasons: (1) The morphological analysis and word-matching model used in this study are simple systems. (2) The methodology functions as a universal approach that transcends language-specific elements. (3) Some shortcomings in machine scoring can be addressed by updating the list of correct words and dictionaries. As a future direction, we aim to apply the approach used in this study to conduct scoring in other languages and pursue further advancements.

Additionally, as a future development, we are considering using large-scale language models for machine scoring. Natural language processing technology has evolved from simple word matching and counting to polarity detection and negation detection and further to feature extraction of sentences while maintaining context. Currently, large-scale language models have considerably advanced with the use of attention mechanisms. While word matching and counting are very basic but reliable, it is difficult to prove the reliability of large-scale language models. Depending on the content we want the machine to score, it is necessary to use these technologies appropriately. With current technology, this differentiation is possible, whereas in the future, more accurate machine scoring can be expected. This point should be a topic of future research.

### Limitations

This is a pilot study, and the sample size may be insufficient for statistical examination of the differences between human and machine scoring. However, we can discuss the discrepancies between them quantitatively and qualitatively. In this study, we demonstrate that the examination and evaluation of qualitative discrepancies can serve as a simulation of calibration algorithms and dictionaries. We have yet to examine the generalizability of the algorithm used in this study and its results. Adapting the present results to other tests is a topic for future research.

Additionally, the current program has a considerable limitation in that it cannot detect whether a term is used in a positive or negative context. For example, if a participant responds with “no heart murmur,” the program can only identify the presence of the term “heart murmur” and cannot understand that the context is negative. This limitation highlights the challenge of evaluating medical accuracy at the word level using machine scoring. The current program is somehow limited in determining the medical correctness of free-text responses. As a future improvement, these contextual interpretation issues are likely to be solved by incorporating next-generation natural language processing technologies such as the Generative Pre-trained Transformer model. This should be a subject for future research.

### Conclusion

We mechanically scored the free-text responses to the GM-ITE patient-reproduction video-based questions with simulated patients using a simple program of morphological analysis and matching with dictionaries and correct words. We found that machine scoring was comparable to human scoring in 39 responses to 265 questions. We found discrepancies between human and machine scoring in 7.2% of the cases. While machine scoring in this study had the rigor to avoid human error, it could not detect unprepared words and was unsuitable for parsing sentences. Machine scoring requires a simple program and calibration and can potentially reduce the cost of scoring free-text responses.

## Supplementary material

10.2196/52068 Multimedia Appendix 1 Correct words for each question.

10.2196/52068 Multimedia Appendix 2 Words added to the user dictionary.

10.2196/52068 Multimedia Appendix 3 Analysis of discrepancies between human and machine scoring in Japanese and English.

## Abbreviations

CBT: computer-based test
GM-ITE: General Medicine In-Training Examination
JAMEP: Japan Institute for Advancement of Medical Education Program
SBAR: Situation, Background, Assessment, and Recommendation
