# Supplementary material for: Evaluation of a Computer-Based Morphological Analysis Method for Free-Text Responses in the General Medicine In-Training Examination: Algorithm Validation Study
Source: JMIR Med Educ. 2024 Dec 5;10:e52068. doi: 10.2196/52068 (PMC11637224; doi:10.2196/52068)
Supplement: Multimedia Appendix 1 [file mededu-v10-e52068-s001.docx]

Table S1. Correct words for each question

| Question number | Answers in Japanese |
| --- | --- |
| 3-1 | 吉永誠一 |
| 3-2 | 45 歳, 45 才 |
| 3-3 | 男性, 男 |
| 3-4 | 急性肺血栓塞栓症, 急性肺塞栓, 急性肺塞栓症, 肺血栓塞栓症, 肺塞栓症, 肺塞栓, 肺動脈塞栓症, PE |
| 3-5 | 意識消失, 失神, 意識消失発作, 意識を消失, 意識を失う |
| 4-1 | 骨折, 右下腿の骨折, 右下腿骨折, 下腿骨折, 右下肢骨", 下肢骨折" |
| 4-2 | アムロジピン |
| 4-3 | (deleted) |
| 4-4 | 数分, 10 秒, 10 秒間 |
| 5-1 | 頻呼吸, 呼吸数の増加, 呼吸数が多 |
| 5-2 | 外頸静脈怒張, 外頚静脈怒張, 頸静脈怒張, 頚静脈怒張, 頸静脈の怒張, 頚静脈の怒張 |
| 5-3 | 収縮期雑音, 収縮期, 心雑音, 雑音 |
| 5-4 | IIp 亢進, Iip 亢進, IIp 亢進, S2 亢進, II 音の亢進, II 音の亢進,  II 音亢進, 2 音亢進, ２音亢進, 2 音の亢進, ２音の亢進 |
| 5-5 | 骨折 |
| 6-1 | 心電図, ECG, 心電図検査 |
| 6-2 | 心エコー, 心 echo, 心臓超音波, TTE, 心臓超音波検査, 心エコー検査, 心臓エコー |
| 6-3 | 造影 CT |
| 6-4 | 抗凝固薬 |
| 6-5 | 血栓溶解薬 |
