# Supplementary material for: Evaluation of a Computer-Based Morphological Analysis Method for Free-Text Responses in the General Medicine In-Training Examination: Algorithm Validation Study
Source: JMIR Med Educ. 2024 Dec 5;10:e52068. doi: 10.2196/52068 (PMC11637224; doi:10.2196/52068)
Supplement: Multimedia Appendix 2 [file mededu-v10-e52068-s002.docx]

Table S2. Words added to the user dictionary

| Words added in Japanese | Cost | Parts of speech (POS) | POS  subcategory 1 | POS  subcategory 2 | POS  subcategory 3 |
| --- | --- | --- | --- | --- | --- |
| 吉永誠一 | 1 | noun | proper | person’s name | firstname |
| 吉永誠一 | 1 | noun | proper | person’s name | surname |
| 吉永誠一 | 1 | noun | general |  |  |
| 意識を消失 | 1 | noun | suffix (verb) |  |  |
| 静脈の怒張 | 1 | noun | general |  |  |
| 静脈怒張 | 1 | noun | general |  |  |
| 頸静脈の怒張 | 1 | noun | general |  |  |
| 頸静脈の怒張 | 1 | noun | general |  |  |
| 頸静脈怒張 | 1 | noun | general |  |  |
| 頸静脈怒張 | 1 | noun | general |  |  |
| 外頸静脈の怒張 | 1 | noun | general |  |  |
| 外頸静脈の怒張 | 1 | noun | general |  |  |
| 外頸静脈怒張 | 1 | noun | general |  |  |
| 外頸静脈怒張 | 1 | noun | general |  |  |
| IIp 亢進 | 1 | noun | general |  |  |
| Iip 亢進 | 1 | noun | general |  |  |
| IIp 亢進 | 1 | noun | general |  |  |
| II 音の亢進 | 1 | noun | general |  |  |
| II 音亢進 | 1 | noun | general |  |  |
| II 音の亢進 | 1 | noun | general |  |  |
| 2 音の亢進 | 1 | noun | general |  |  |
| 2 音亢進 | 1 | noun | general |  |  |
| ２音の亢進 | 1 | noun | general |  |  |
| S2 亢進 | 1 | noun | general |  |  |
| 心臓エコー | 1 | noun | general |  |  |
| Words added in Japanese | Cost | POS | infl_type | infl_form | base_form |
| 意識を失う | 1 | verb | godan_wa_a_column_change_t | base | 意識を失う |
| 意識を失わ | 1 | verb | godan_wa_a_column_change_t | irrealis | 意識を失う |
| 意識を失お | 1 | verb | godan_wa_a_column_change_t | irrealis_u | 意識を失う |
| 意識を失い | 1 | verb | godan_wa_a_column_change_t | continuative | 意識を失う |
| 意識を失っ | 1 | verb | godan_wa_a_column_change_t | continuative_ta | 意識を失う |
| 意識を失え | 1 | verb | godan_wa_a_column_change_t | conditional | 意識を失う |
| 意識を失え | 1 | verb | godan_wa_a_column_change_t | imperative_e | 意識を失う |
