# Supplementary material for: Evaluation of a Computer-Based Morphological Analysis Method for Free-Text Responses in the General Medicine In-Training Examination: Algorithm Validation Study
Source: JMIR Med Educ. 2024 Dec 5;10:e52068. doi: 10.2196/52068 (PMC11637224; doi:10.2196/52068)
Supplement: Multimedia Appendix 3 [file mededu-v10-e52068-s003.docx]

Table S3. Analysis of discrepancies between human and machine scoring in Japanese and English

| ID | Question number | Answers in Japanese and English | Human | Machine | Reason for the discrepancy |
| --- | --- | --- | --- | --- | --- |
| #1 | 3-5 | 予兆なく意識障害で倒れた４５歳男性です。  He is a 45-year-old male who collapsed with a disturbance of consciousness^b^ without signs. | correct | mistake†^a^ | “Disturbance of consciousness” is an incorrect answer. |
| #2 | 3-5 | *目の前が真っ暗になりたおれた*  He *was plunged into darkness and fell.*^c^ | correct† | mistake | This is a *sentence*. |
| #3 | 3-5 | トイレでの眼前暗黒**館**自覚後、転倒された患者様です。  This patient fell after **feeling**^d^ dimmed vision in the restroom. | correct | mistake† | The correct word list does not include “dimmed vision”; it is also **Misspelled.** |
| #4 | 3-5 | トイレに行こうと立ち上がったら倒れた。その時に眼前暗黒感があった。  He *stood up to go to the restroom and fell*. He had a dimmed vision. | correct† | mistake | The correct word list does not include “dimmed vision,” and this is a *sentence*. |
| #5 | 3-5 | *立ちあがった際に目の前が真っ暗になり転倒し*  *When he stood up, he was plunged into darkness and fell.* | correct† | mistake | This is a *sentence*. |
| #6 | 4-1 | 転倒し右足関節付近の骨折を指摘  He fell and was noted to have a fracture near his right ankle joint. | mistake | correct† | Human error |
| #7 | 4-1 | 右足を怪我されておりギプス固定されております  He has an injury to his right leg, which is in a cast. | correct | mistake† | “Injury” is an incorrect answer. |
| #8 | 4-2 | 内服歴：アムロジピン  Medications: Amlodipine | mistake | correct† | Human error |
| #9 | 4-2 | 高血圧の既往歴があり、アムロジピン服薬中です。  He has hypertension and takes Amlodipine. | mistake | correct† | Human error |
| #10 | 4-3 | 意識消失がありすぐ改善した  He had a blackout, which improved quickly. | correct† | mistake | “Improved quickly” is not in the dictionary. |
| #11 | 5-2 | **軽**静脈怒張や収縮期雑音から、弁膜症も鑑別に挙げられます。  Valvular disease is also a differential based on **jugular** vein distension and systolic murmur. | correct | mistake† | **Misspelled**. |
| #12 | 6-2 | エコー検査にて、まず肺塞栓・一過性脳虚血の否定を行う。  Echography is first used to deny pulmonary embolism and transient cerebral ischemia. | correct | mistake† | “Echography” is an incorrect answer. |
| #13 | 6-2 | エコーを行い弁運動を評価すべきだと思います。  I should perform an echo to evaluate valve motion. | correct | mistake† | “Echo” is an incorrect answer. |
| #14 | 6-2 | エコーで EF が保たれているかなどの精査が必要。  He needs a close examination, including an echo, to see if the EF is preserved. | correct | mistake† | “Echo” is an incorrect answer. |

^a^† means that it is a correct decision.

^b^Underlined words indicate that the answer is incorrect, not on the list of correct answers, or not included in the dictionary.

^c^ Italics indicate that the original Japanese sentence cannot be broken down into words and is expressed as a sentence.

^d^The bold text indicates a misspelling.
